# Mitochondrial Genome Variation in Breast Muscle of Tibetan Fragrant Chickens Across an Altitudinal Gradient and Its Association with Meat Quality

**DOI:** 10.3390/ani16213410

**Published:** 2026-10-29

**Authors:** Zengwen Huang, Jing Wang, Jun Li, Dayong Si, Zengpeng Lü, Gang Shu, Zhiqiang Hu, Chaoyun Yang

**Affiliations:** 1 College of Animal Science, Xichang University, Xichang 615013, China; xndaxue@126.com (Z.H.); slnee.5703@163.com (J.W.); lijun@xcc.edu.cn (J.L.); zhiqianghu0624@163.com (Z.H.); 2 College of Animal Science and Technology, China Agricultural University, Beijing 100193, China; dayong@cau.edu.cn; 3 College of Veterinary Medicine, Sichuan Agricultural University, Chengdu 611130, China; dyysg2005@sicau.edu.cn

**Keywords:** Tibetan fragrant chicken, mitochondrial genome, high-altitude adaptation, maternal haplotype, heteroplasmy, meat quality

## Abstract

This study aimed to characterize the mitochondrial (MT) genome of Tibetan fragrant chickens along an altitudinal gradient and to relate it to meat quality. Eighteen 150-day-old male chickens were selected from three groups (LX, 1500 m; MX, 2800 m; HX, 3500 m; n = 6 each; six birds were randomly chosen from 30 reared per group) and subjected to whole-genome resequencing (reference: *Gallus gallus* GCA_000002315.5). Ninety MT variants (85 polymorphic; transition/transversion 83:7) were identified, comprising 58 coding (42 synonymous, 16 non-synonymous), 20 D-loop/non-coding, 10 rRNA and 2 tRNA sites; no MT insertions/deletions or structural variants were found. The 18 birds clustered into three maternal lineages (A, B, C) that were only partially structured by altitude (A more frequent at low altitude; B at mid and high altitude). A neighbour-joining tree with 12 *Gallus* mitogenomes placed the population close to *Gallus gallus gallus* and Chinese local breeds. Fifteen heteroplasmic sites were detected, with 16S rRNA nt3946 (G/C/A) heteroplasmic in all birds. Relative mtDNA copy number did not differ among groups (*p* = 0.52). Meat quality varied with altitude (highest protein and lowest fat at 2800 m; highest inosine monophosphate at 3500 m) but not with maternal lineage, suggesting that the observed phenotypic differences are associated mainly with altitude of rearing rather than with maternal mitochondrial lineage, and that the MT genome contributes standing genetic variation rather than a direct functional basis for these differences.

## Simple Summary

Tibetan fragrant chickens live at high altitudes where oxygen is scarce. Mitochondria, the powerhouses of cells, may help them cope with low oxygen. The whole genome of breast muscle from 18 male chickens raised at three altitudes (1500, 2800 and 3500 m) was sequenced, and the mitochondrial genome was analysed. Ninety mitochondrial variants were found, most of which were shared among birds, and no insertions, deletions or structural changes were detected. The birds grouped into three maternal lineages (A, B and C) that were only weakly related to altitude. Fifteen sites carried mixed (heteroplasmic) variants, and one site in the 16S rRNA gene was mixed in every bird. Mitochondrial DNA copy number did not differ among altitudes. In contrast, meat quality changed mainly with altitude (higher protein and lower fat at 2800 m; higher umami-related inosine monophosphate at 3500 m) rather than with maternal lineage. The results suggest that high-altitude adaptation in these chickens is driven mainly by nuclear genes and the environment, whereas the mitochondrial genome mainly contributes standing genetic diversity.

## 1. Introduction

The Tibetan fragrant chicken is a local breed that was formed on the Qinghai–Tibet Plateau through long natural selection and household domestication, and it is one of the few poultry breeds that can complete its life cycle at altitudes of about 2200–4100 m [1,2]. It has a compact body, keen foraging ability, cold and roughage tolerance and strong stress resistance, and its muscle is rich in amino acids and inosine monophosphate (IMP), giving an intense umami flavour and high nutritional value. Muli Tibetan Autonomous County (Sichuan) lies at the eastern margin of the Hengduan Mountains, with an altitudinal span of more than 4400 m; its long geographic isolation may have produced features distinct from those of other Tibetan chickens [3], and the breed already mounts distinct molecular responses to chronic hypoxia during development [4].

Mitochondria are the core organelles of aerobic respiration, and the mitochondrial genome (mtDNA) is a small, circular, maternally inherited molecule that evolves rapidly, occurs at high copy number and frequently carries heteroplasmy, making it a widely used marker for maternal structure and adaptive evolution [3]. It encodes 13 oxidative phosphorylation (OXPHOS) subunits, two rRNAs, 22 tRNAs and a non-coding control region (D-loop). Under hypoxia, respiratory-chain efficiency and energy supply directly determine the ability to cope with low oxygen, so respiratory-chain genes are candidate loci for hypoxia adaptation [5]. Because the respiratory-chain complexes are encoded by both nuclear and mitochondrial genes, coding variation in the mitochondrial subunits may contribute to functional adaptation of these complexes [5,6]. In muscle, which has very high and variable mtDNA content, heteroplasmy and copy number add further layers of mitochondrial variation that can be described alongside sequence variation [6].

Chicken mtDNA haplogroups (A-E and related lineages) reflect maternal origin, geography and artificial selection [7], and D-loop surveys show that native Chinese chicken breeds retain high maternal diversity [8]. In Tibetan chickens, MT-CO3 haplotypes and variants in MT-COI and ATP6 have been associated with high-altitude adaptation, supporting a possible mitochondrial contribution to hypoxia tolerance [1,2]. Beyond sequence variation, heteroplasmy and mtDNA copy number are additional layers of mitochondrial variation [6], and mitochondrial sequence variation has been widely used to characterise the genetic diversity of domestic chickens [8].

Despite this progress, most mitochondrial studies of Tibetan chickens have focused on a single gene or the D-loop and on Tibetan populations, and a systematic integration of sequence variation, maternal structure, heteroplasmy and copy number along an altitudinal gradient is still lacking [2,3]. The mitochondrial features of the Muli population and their relationship to meat quality have not been characterized. The present study therefore analysed whole-genome resequencing data from the breast muscle of Tibetan fragrant chickens at three altitudes (1500, 2800 and 3500 m) in Muli County, with the objectives to (i) characterize the MT variant spectrum, (ii) define and phylogenetically place maternal lineages, (iii) describe heteroplasmy, (iv) estimate relative mtDNA copy number and (v) relate these features to phenotypic and meat-quality variation, and to test whether they track altitude [2,3].

From an ecological and production standpoint, understanding the mitochondrial genetic makeup of the Muli population is also practically relevant. Local chicken breeds on the plateau are increasingly exposed to cross-breeding with commercial lines, and documenting maternal lineages provides a baseline against which genetic erosion can be monitored. At the same time, muscle mitochondrial features such as heteroplasmy and copy number may influence oxidative capacity and, indirectly, meat-quality traits that are important for the premium market of Tibetan fragrant chicken [2,3]. Establishing whether such features vary with altitude therefore has both conservation and product-quality implications.

More broadly, comparative genomic studies of high-altitude human populations, together with experimental avian models, implicate the hypoxia-inducible factor (HIF) pathway and genes such as EPAS1 in adaptation to low oxygen [9,10,11,12], and structural genomic features such as polymorphic transposable elements have recently been proposed to contribute to high-altitude adaptation [13]. These observations frame the present study: if mitochondrial variation contributes to adaptation in this breed, it should leave detectable signatures in maternal lineage structure, heteroplasmy or copy number along the altitudinal gradient.

## 2. Materials and Methods

### 2.1. Animals and Experimental Design

The experiment was conducted with Tibetan fragrant chickens at a demonstration farm in Muli County, Sichuan Province, China (eastern margin of the Hengduan Mountains). Only male chickens were used in this study, and no breeding (maternal) flock was maintained by the authors; the experimental birds were obtained as day-old chicks from this demonstration farm. The chicks were hatched at middle altitude (2800 m) and reared there until 30 days of age, after which 30 birds were transferred to the low-altitude site (low-altitude group, LX, 1500 m) and 30 birds to the high-altitude site (high-altitude group, HX, 3500 m), while a further 30 birds were kept at the middle-altitude site (middle-altitude group, MX, 2800 m). All three groups were then reared to 150 days of age under the same management regime; because the LX and HX groups were moved to their final altitudes at 30 days of age, they were acclimated to these altitudes for approximately 120 days (from 30 to 150 days of age) before sampling, A single rearing site was deliberately used for each altitude so that management practice, stocking density, feeding regimen and animal-handling personnel remained constant within a group; this minimizes management-related random error and avoids confounding of the altitudinal contrast by differences in husbandry style. and each group was sampled at the same site and altitude at which it was reared. All groups were fed the same commercial diet, and water and feed were provided ad libitum, ensuring a consistent rearing background across groups. Lighting followed the natural local photoperiod at each altitude, and no supplemental lighting was provided. At 150 days of age, six male birds with similar body weight and health status were randomly selected from each group and slaughtered, giving 18 birds in total; random selection was intended to reduce sampling bias and to ensure comparability among groups. After slaughter, breast muscle (pectoralis major) was collected rapidly, snap-frozen in liquid nitrogen and stored at −80 °C until sequencing and phenotypic analysis. All procedures involving animals were carried out in accordance with the guidelines for the care and use of agricultural animals in research and teaching, and the animal study protocol was approved by the Ethics Committee of Xichang University (approval number xcc20250918).

### 2.2. Whole-Genome Resequencing and Quality Control

Genomic DNA was extracted from breast muscle. DNA was randomly fragmented to approximately 350 bp with a Covaris instrument (Covaris, Inc., Woburn, MA, USA); ends were repaired with T4 DNA polymerase and polynucleotide kinase, a single adenine was added to the 3′ ends, and Illumina sequencing adapters were ligated with T4 DNA ligase (New England Biolabs, Ipswich, MA, USA). Fragments were size-selected with Agencourt SPRIselect magnetic beads (Beckman Coulter, Brea, CA, USA; cat. no. 2358413) using double size selection, amplified by PCR with a high-fidelity polymerase, and quantified with a Qubit 3.0 fluorometer (Thermo Fisher Scientific, Waltham, MA, USA). Insert size was checked on an Agilent 5400 system (Agilent Technologies, Santa Clara, CA, USA), and the effective library concentration was determined by qPCR (1.5 nM). Libraries were sequenced on an Illumina NovaSeq 6000 platform (Illumina, Inc., San Diego, CA, USA) in paired-end mode (PE150) to an average depth of approximately 4× per individual. The reference genome was the chicken assembly *Gallus gallus* GCA_000002315.5 (GRCg6a) [14]. Raw reads were processed with standard quality control: paired reads were discarded if either read contained adapter contamination (>10 nucleotides aligned to the adapter, allowing ≤10% mismatches), if more than 10% of bases were ambiguous (N), or if more than 50% of bases had a Phred quality < 5. Clean reads were aligned to the reference genome with BWA (mem -t 4 -k 32 -M, (v0.7.17)), and PCR duplicates were removed with SAMtools (rmdup, v1.16;). Alignment statistics—mapping rate, mean sequencing depth, and the proportions of the genome covered at ≥1× and ≥4×—and base-quality metrics (error rate, Q20 and Q30) were calculated with SAMtools. All samples were processed with the same pipeline so that quality metrics were directly comparable among the three altitude groups. A mean depth of approximately 4× was chosen to allow reliable detection of high-confidence sites in a small number of individuals while keeping the analysis focused on the mitochondrial genome, which is present at very high copy number per cell and is therefore covered comparatively well even at modest nuclear depth.

### 2.3. Variant Calling and Mitochondrial Variant Extraction

Single-nucleotide polymorphisms (SNPs) and small insertions/deletions (InDels) were called with SAMtools mpileup (-C 50 -m 2 -F 0.002 -d 1000; (v1.16)); this caller is widely used for variant detection in non-human species, and its performance relative to GATK HaplotypeCaller has been benchmarked [15]. To reduce the SNP error rate, variants were retained only when supported by at least 4 reads and with a mapping quality (MQ) ≥ 20; InDels shorter than 50 bp were called from the same alignments and filtered with QD < 2.0, FS > 200.0 and ReadPosRankSum < −20.0. Structural variants (SVs) were detected with BreakDancer (v1.4.4), and calls supported by fewer than 2 paired-end reads were removed; copy-number variants (CNVs) were detected with CNVnator (v0.3;-call 100). All variants were functionally annotated with ANNOVAR (2015Dec14) using the UCSC known-gene models [16]. Given the moderate sequencing depth and the known behaviour of variant callers in non-model species, filtering thresholds were deliberately conservative [15]. Because the reference genome contains a mitochondrial contig, mitochondrial variants were extracted using the chromosome identifier “MT” (length 16,775 bp). The extracted variants were mapped to transcript/mRNA, coding-sequence (CDS), rRNA, tRNA and D-loop regions on the basis of the reference annotation, and the effect of each coding variant (synonymous or non-synonymous) was determined using the vertebrate mitochondrial genetic code; the corresponding amino-acid substitutions were also recorded. To control false positives, only variants that passed the depth and quality filters were retained, and for multi-allelic sites the depth of each allele was recorded separately. Sites that failed the mapping-quality or depth thresholds were removed, and the mitochondrial variants retained for analysis were checked manually against the reference sequence to exclude obvious mis-alignments, particularly in the control region, where homopolymer runs and short repeats can generate spurious calls. Each retained variant was required to have a total depth of at least 4 reads and a genotype quality sufficient to distinguish the reference and alternative alleles. It was also taken into account that the reference chicken assembly contains regions that are difficult to assemble, and calls in such regions were therefore treated conservatively [17].

### 2.4. Haplotype and Phylogenetic Analyses

A pairwise difference matrix was constructed from the alleles at the 90 MT variant sites, treating an allele frequency of ≥0.5 as the variant state and lower frequencies as the reference state, and maternal lineages were defined by hierarchical (UPGMA) clustering of this matrix. For phylogenetic placement, 12 complete mitochondrial genomes of *Gallus* spp. were downloaded from NCBI as references. The MT sequence of each of the 18 samples was reconstructed by applying its own variants to the reference sequence, and the reconstructed sequences were compared with the reference mitogenomes; a neighbour-joining (NJ) tree was then constructed, and the number of differences between each sample and each reference was counted [18]. The reconstructed sequences were also compared with the reference mitogenomes to check for numt-like signatures.

### 2.5. Heteroplasmy Analysis

Heteroplasmy was assessed from per-sample allele frequencies calculated from the allele-depth (AD) field, with the variant allele frequency (VAF) defined as alt depth divided by the sum of reference and alt depths. Sites with 0.05 < VAF < 0.95 and adequate sequencing depth were recorded as heteroplasmic. For representative heteroplasmic sites, the genotype, total depth (DP) and allele depths (AD) of each sample were inspected at read level, and a site was accepted as heteroplasmic only when the minor allele was supported by multiple reads, so as to reduce false heteroplasmy caused by sequencing error [19]. For each candidate site it was additionally required that the minor allele be supported on both strands where possible, and genotype calls from low-depth samples were treated with particular caution.

### 2.6. mtDNA Copy Number Estimation

The ratio of the mean MT depth to the mean nuclear genome depth was used as a proxy for relative mtDNA copy number (CN = MT depth/nuclear depth), a procedure that uses the same sequencing data and largely cancels differences in sequencing effort among individuals [20]. This ratio-based approach is widely used for relative quantification from sequencing data and is more robust to differences in library size than absolute depth, although it does not recover absolute molecule numbers [20]. Group means were compared as described below.

### 2.7. Phenotype Measurement and Statistical Analysis

Body weight, total caecum length and the weights of the heart, lung, spleen, liver and bursa of Fabricius were recorded. Meat-quality traits (132 indices in total) were measured, including moisture, crude protein, crude fat, individual and total amino acids, fatty acids, volatile flavour compounds and nucleotides such as inosine monophosphate (IMP), guanosine monophosphate (GMP) and uridine monophosphate (UMP). Data are presented as mean ± standard deviation. Groups were compared by one-way analysis of variance or by the Kruskal–Wallis test when the normality or variance assumptions were not met, and To control the false-discovery rate across the many traits measured, Benjamini–Hochberg correction was applied within the meat-quality trait set, and adjusted q values are reported alongside unadjusted *p* values. Effect sizes and their 95% confidence intervals were calculated for the main comparisons, and differences were considered significant at *p* < 0.05 (two-sided). The association between mitochondrial variation and phenotype was analysed in an exploratory manner because of the sample size, and trait values are reported at the group level. The use of a single sampling altitude per group means that altitude and location cannot be fully separated in this design, and this should be kept in mind when interpreting the phenotypic results.

## 3. Results

### 3.1. Sequencing Quality and Mapping

The 18 samples yielded high-quality data: Q30 ranged from 95.96% to 96.75% and GC content from 43.34% to 45.09%. The mean mapping rate was 95.03%, the mean sequencing depth ranged from 2.99× to 4.69×, and the mean coverage at ≥1× and ≥4× was 88.51% and 32.23%, respectively (Table 1). One high-altitude sample, HX1, showed a notably lower mapping rate (62.94%) and lower coverage (71.95% at ≥1×) than the others, most likely reflecting individual library or sample quality; its variants should therefore be interpreted cautiously, and this sample did not affect the population-level conclusions. Apart from HX1, all samples had mapping rates above 96% and ≥1× coverage above 88%, and the GC content was uniformly within a narrow range, indicating that the libraries were of comparable composition and that the dataset provided uniform coverage of the mitochondrial genome. The number of clean reads per sample was of the same order across groups, so downstream comparisons of depth and coverage were not driven by gross differences in sequencing output.

### 3.2. Mitochondrial Variant Spectrum

**Figure 1:**
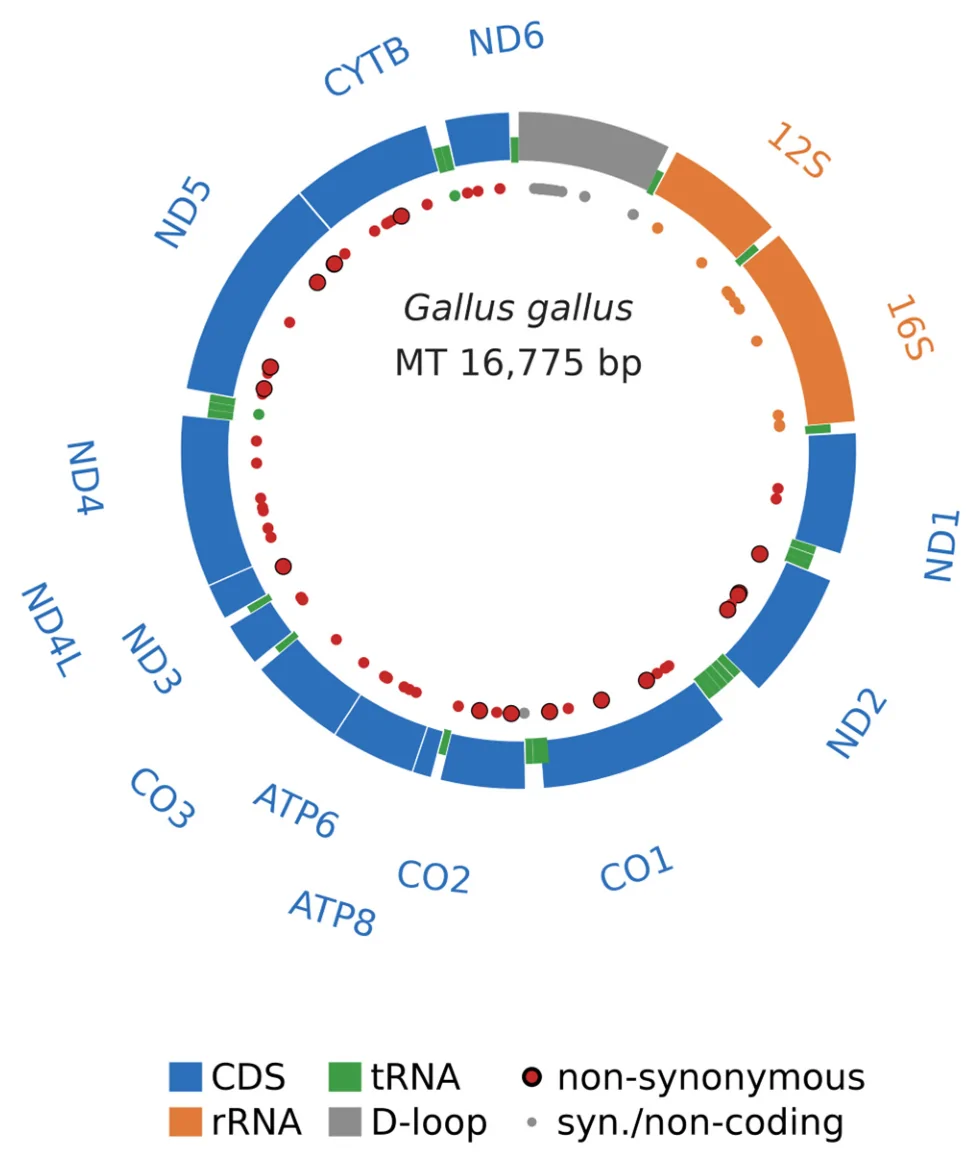
Circular map of the mitochondrial genome of the Tibetan fragrant chicken (16,775 bp) showing gene organization and the distribution of the 90 variant sites. Genes are colour-coded by type: blue, protein-coding genes (CDS); orange, rRNA; green, tRNA; grey, D-loop/control region. Each point on the inner ring represents one variant site; large outlined dots denote non-synonymous substitutions, whereas small filled dots denote synonymous or non-coding variants. Gene abbreviations follow the standard vertebrate mitochondrial nomenclature.

Using the GRCg6a MT sequence (16,775 bp) as the reference, 90 MT variant sites were identified, of which 85 were polymorphic within the population (carried by 1–17 of the 18 birds). Transitions predominated (83 transitions versus 7 transversions; transition/transversion ratio ≈ 11.9), consistent with the substitution bias of mitochondrial genomes. By region, 58 sites were located in the coding sequence, 20 in the D-loop or other non-coding regions, 10 in rRNA genes and 2 in tRNA genes; among the coding sites, 42 were synonymous and 16 were non-synonymous (Table 2). No MT InDels or structural variants were detected, indicating that variation in the MT genome of this population is dominated by point mutations and is structurally conserved. The ratio of non-synonymous to synonymous coding variants was approximately 0.38, below 1, suggesting that purifying selection has acted on the coding genes overall. The sites were distributed across the whole mitochondrial genome (Figure 1), and the great majority were carried by more than one bird, which is consistent with the population sharing a common maternal pool; only a minority of sites were private. The transition/transversion ratio (≈11.9) is characteristic of animal mitochondrial DNA and argues against systematic sequencing artefacts, which would be expected to generate a higher proportion of transversions. Among the polymorphic sites, the number of birds carrying the non-reference allele ranged from 1 to 17, with most sites shared by several individuals.

The 16 non-synonymous substitutions were distributed among respiratory-chain genes, including ND2 (nt5274 p.Ser12Gly, nt5718 p.Ser160Ala, nt5743 p.Met168Thr, nt5928 p.Leu230Met), CO1 (nt7030 p.Val129Ala, nt7530 p.Arg296Gly, nt8070 p.Phe476Leu), CO2 (nt8464 p.Met45Thr, nt8787 p.Ile153Val), ND4L (nt11364 p.Ala57Thr), ND5 (nt13219 p.Ile50Thr, nt13450 p.Ile127Thr, nt14437 p.Arg456Gln, nt14692 p.Leu541Pro, nt14697 p.His543Asn) and CYTB (nt15535 p.Asp215Asn) (Table 3, Figure 1). These substitutions were concentrated in complex I (ND2, ND4L, ND5) and complex IV (CO1, CO2) of the respiratory chain, with one in complex III (CYTB). Among the respiratory-chain genes, ND5 carried the largest number of non-synonymous sites (five), followed by ND2 (four) and CO1 (three), whereas ND4L, CO2 and CYTB each carried one; no non-synonymous variants were detected in the ATP synthase or ND3/ND4 genes. The coding variants were therefore not confined to a single respiratory-chain complex but were spread across complexes I, III and IV, which encode the main proton-pumping and oxygen-reducing steps of oxidative phosphorylation.

### 3.3. Maternal Haplotypes and Phylogenetic Placement

**Figure 2:**
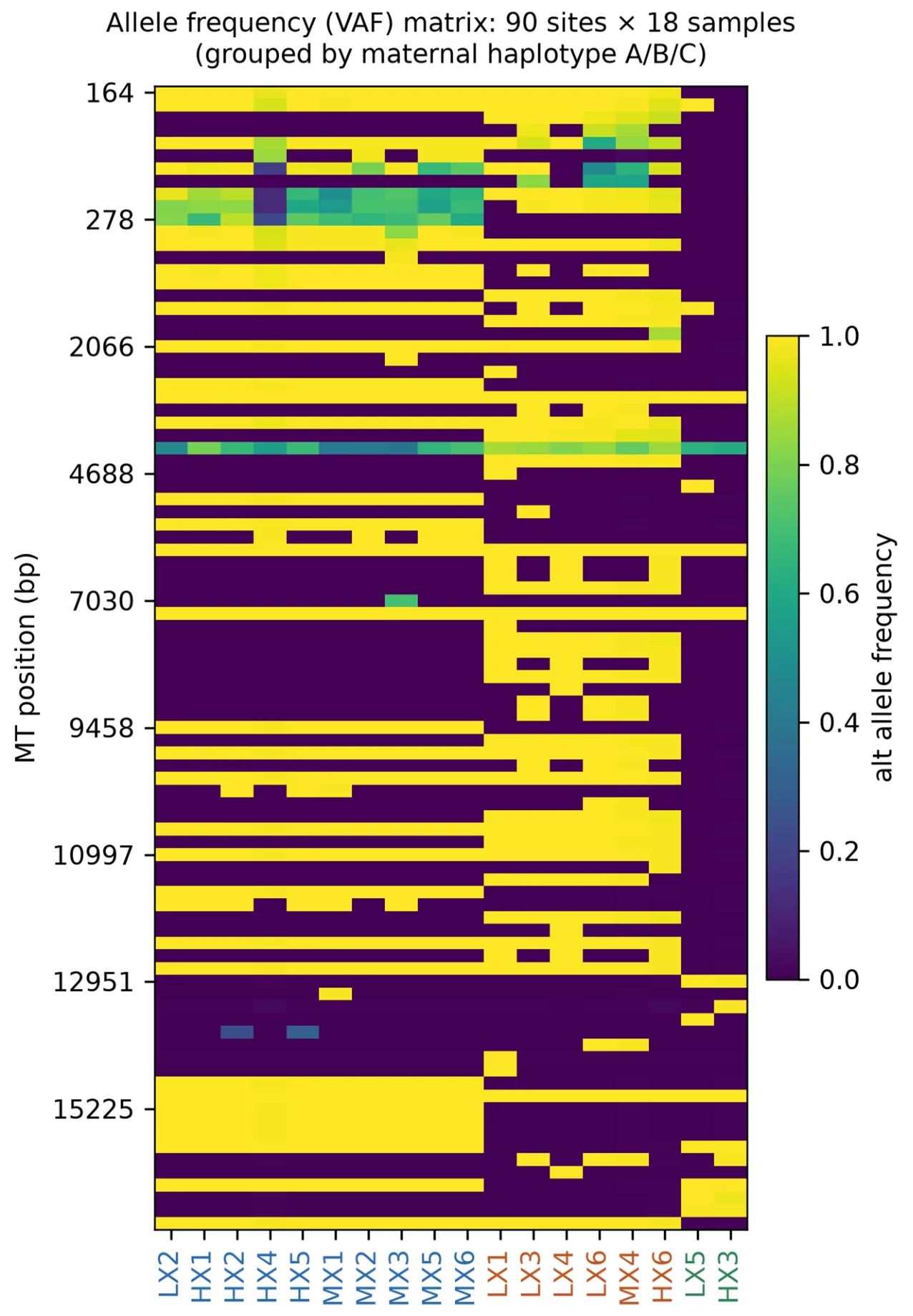
Heat map of allele frequencies (variant allele frequency, VAF) at the 90 mitochondrial variant sites across the 18 samples. Columns are individual birds grouped by maternal lineage (A, B and C; sample labels are coloured accordingly) and rows are mitochondrial positions (bp). The colour scale ranges from 0 (reference-like; dark) to 1 (variant; yellow).

**Figure 3:**
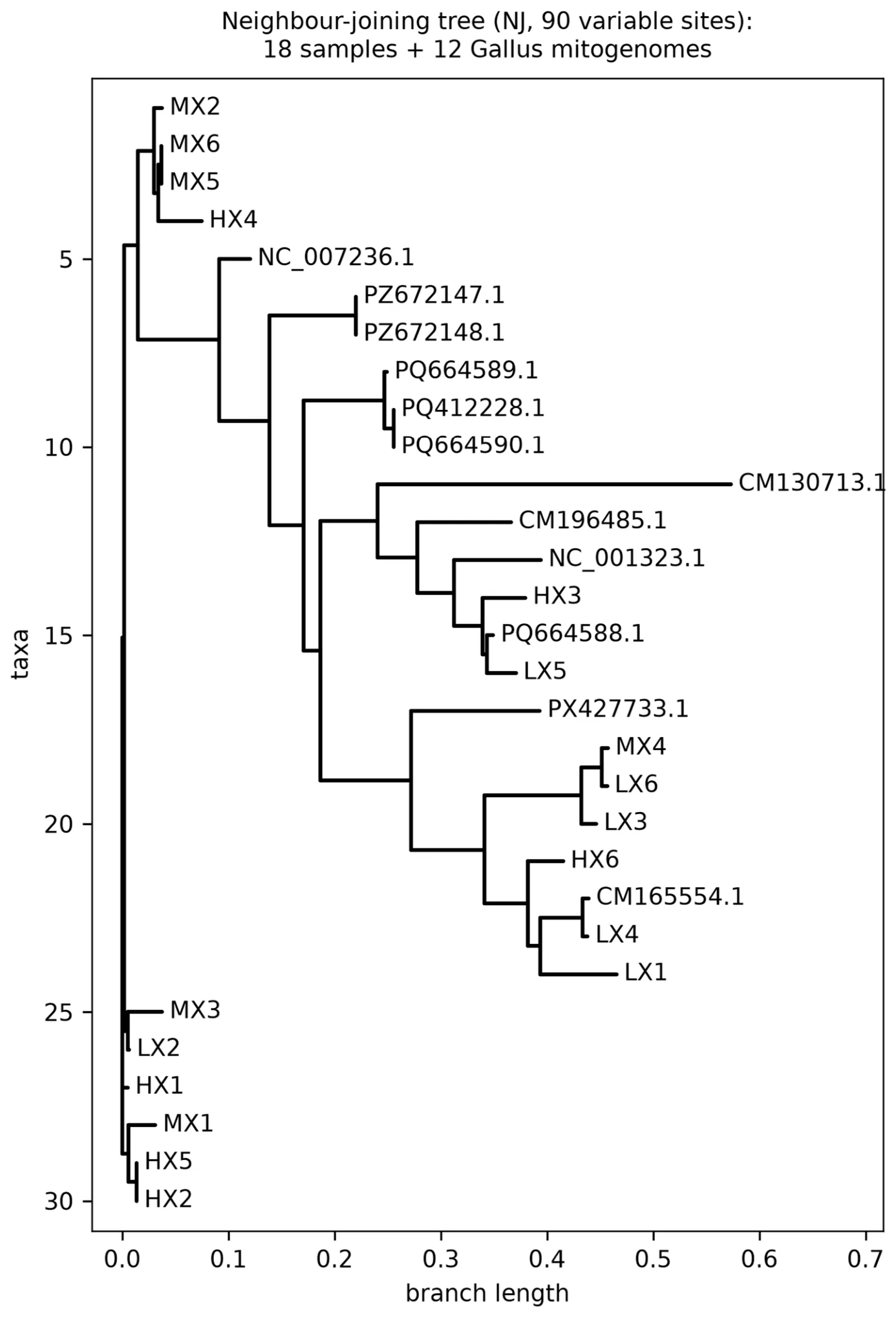
Neighbour-joining tree based on the 90 variable sites, showing the 18 Muli samples and 12 reference *Gallus* mitogenomes. Sample labels: LX1–LX6, MX1–MX6 and HX1–HX6 denote low—(1500 m), middle—(2800 m) and high-altitude (3500 m) birds, respectively. The reference accession numbers correspond to the following breeds or taxa: NC_007236, red junglefowl (*Gallus gallus gallus*); NC_001323, chicken reference (*G. gallus*); CM130713, Silkie; CM165554, Wenshui; CM196485, Wuhua Yellow; PZ672147, Pelung; PZ672148, Sentul; PX427733, Ind/Odi/Gal; PQ412228, TC03; PQ664588, BT02; PQ664589, HO02; and PQ664590, TY02. Branch lengths are proportional to the number of differences per site.

Based on the 90 variant sites, the 18 birds were divided into three maternal lineages. Lineage A comprised LX1, LX3, LX4, LX6, MX4 and HX6; lineage B comprised HX1, HX2, HX4, HX5, MX1, MX2, MX3, MX5, MX6 and LX2; and lineage C comprised LX5 and HX3 (Table 4). Lineage A was more frequent in the low-altitude group (4 of 6 birds), whereas lineage B predominated at middle and high altitudes, indicating mild maternal differentiation along the altitudinal gradient that was nevertheless not fully determined by altitude, since different lineages coexisted within groups and some lineages were shared across altitudes. In the NJ tree constructed with the 12 *Gallus* mitogenomes, the population clustered close to *Gallus gallus gallus* (NC_007236) and to Chinese local breeds; the mean number of differences from the reference sequences was smallest for NC_007236 (21.3 sites) and for PQ664589, PQ664590 and PQ412228 (approximately 26 sites), and larger for the chicken reference NC_001323 (37.2 sites) (Figure 2 and Figure 3). Within lineage B, the largest and most widely distributed lineage, birds from all three altitudes were represented, whereas lineage C was represented by only two birds (LX5 and HX3). The number of pairwise differences among the 18 reconstructed MT sequences was modest, and no sequence was identical to any of the reference mitogenomes, consistent with the Muli population representing a distinct but closely related maternal group. The three lineages were not equally abundant, with lineage B accounting for 10 of the 18 birds and lineage C for only two.

### 3.4. Heteroplasmy

**Figure 4:**
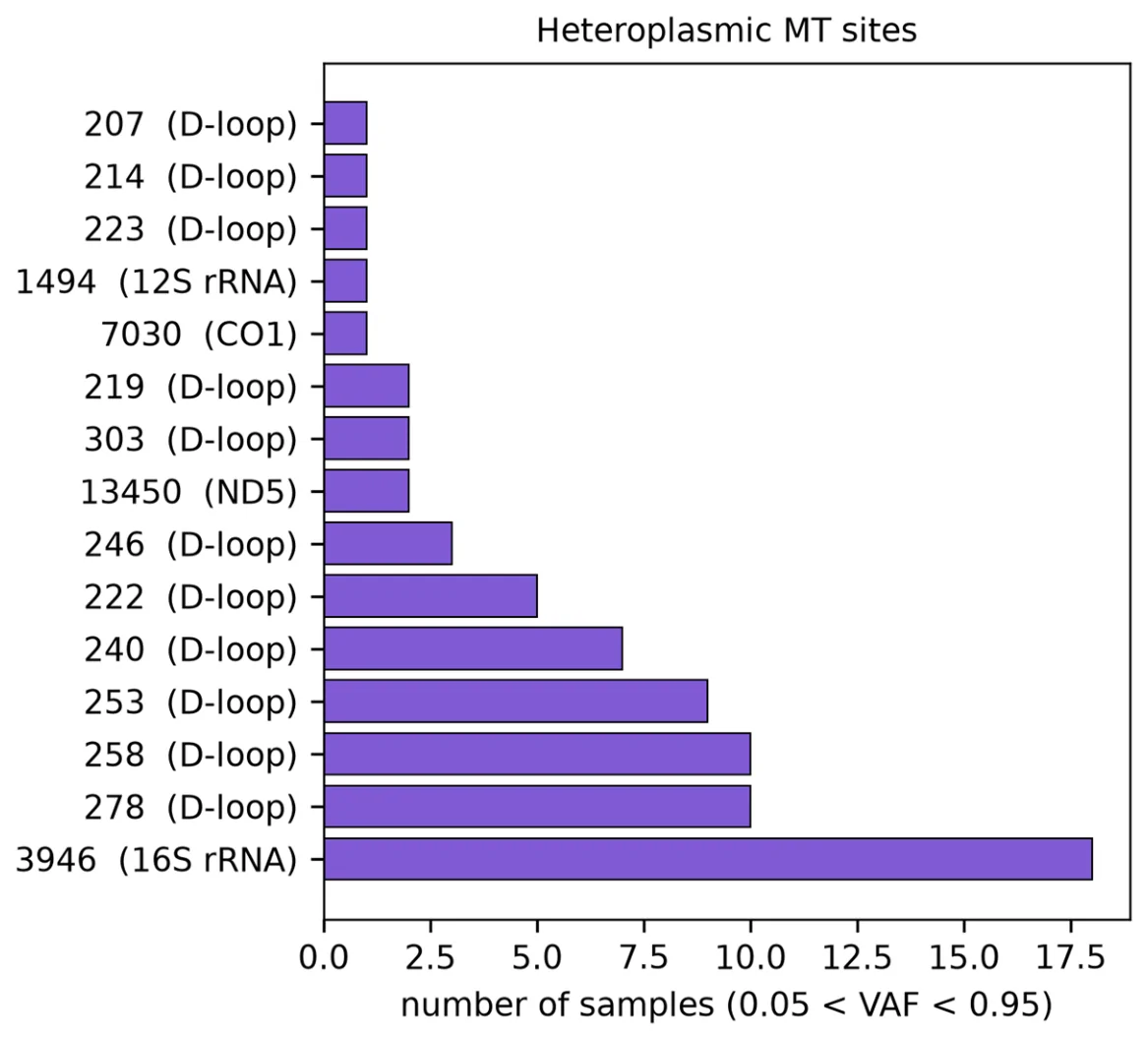
Number of samples carrying each heteroplasmic mitochondrial site (0.05 < VAF < 0.95). The *y*-axis lists the site position and its regional assignment (16S rRNA, D-loop, 12S rRNA, CO1 or ND5); the bar length is the number of samples (out of 18) in which the site was scored as heteroplasmic.

Fifteen heteroplasmic sites (0.05 < VAF < 0.95) were detected. Among them, 16S rRNA nt3946 (reference G; alleles C and A) was heteroplasmic in all 18 birds, with non-reference allele frequencies ranging from 0.55 to 0.87 and strong read-level support (Table 5), making it the most stable heteroplasmic site in the population. Several other sites (nt258, nt278, nt253, nt240 and nt222) showed intermediate frequencies in multiple samples, and nt13450 in ND5 was sample-specific (HX2, VAF = 0.24; HX5, VAF = 0.30). Heteroplasmy calls in low-depth samples, such as MX2 (DP = 15), should be treated with caution (Figure 4). From a functional perspective, most heteroplasmic sites were located in the 16S rRNA and D-loop regions, whereas only a few were in coding regions, indicating that heteroplasmy in this population is concentrated in non-coding and translation-related regions. The heteroplasmic sites were not distributed uniformly across the genome: they were enriched in the 16S rRNA gene and in the D-loop, and coding heteroplasmies were limited to a few sites, with non-reference allele frequencies in the low-to-intermediate range.

### 3.5. mtDNA Copy Number

**Figure 5:**
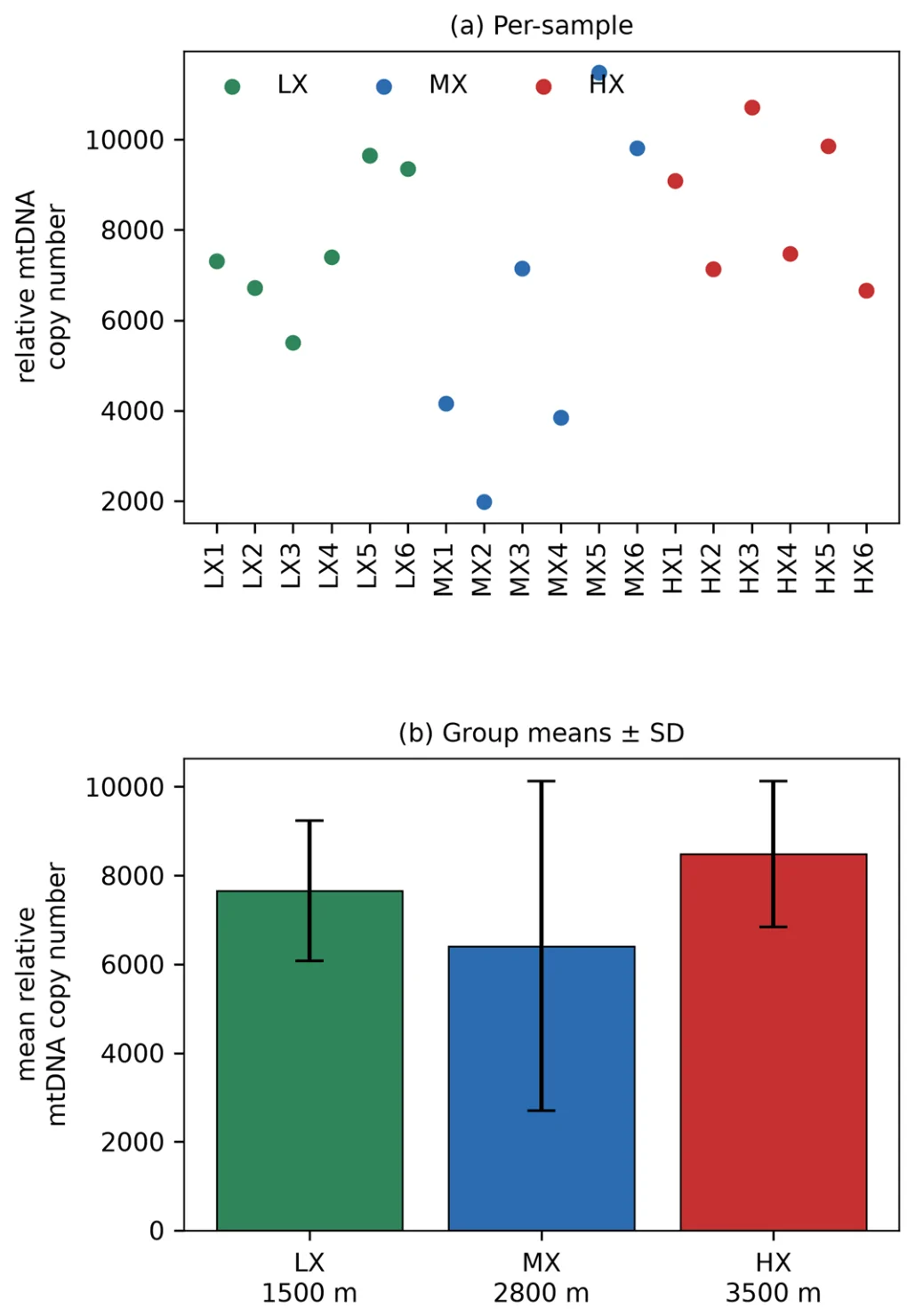
Relative mtDNA copy number (mean MT depth/mean nuclear depth). (**a**) Individual values for all 18 birds, coloured by group (LX, green; MX, blue; HX, red). (**b**) Group means ± SD; the Kruskal–Wallis test showed no significant difference among groups (*p* = 0.52).

The relative mtDNA copy number varied considerably among individuals (MX2 ≈ 1989; MX5 ≈ 11,484). The group means were 7656 for LX, 6405 for MX and 8488 for HX; the difference was not significant (Kruskal–Wallis *p* = 0.52), and no consistent trend with altitude was observed (Table 6, Figure 5). Notably, copy number was not simply proportional to sequencing depth, indicating that it reflects individual and tissue factors rather than technical variation alone. Individual estimates spanned an order of magnitude, from approximately 1989 in MX2 to approximately 11,484 in MX5, and the group medians were close to one another; because copy number was expressed relative to nuclear depth, this variation is unlikely to be explained by differences in sequencing effort alone. Within each group the spread of values was wider than the difference between group means, which further supports the conclusion that altitude had no consistent effect on relative copy number.

### 3.6. Phenotypes and Meat Quality

Heart and lung weights and IMP content increased with altitude (heart: LX, MX and HX = 8.54, 8.23 and 11.02 g; IMP = 775.2, 778.5 and 828.3 mg/100 g), whereas no consistent differences were found when the samples were grouped by maternal lineage (heart: A, B and C = 8.94, 9.41 and 9.48 g), indicating that these traits track altitude rather than MT lineage (Table 7). Meat-quality traits also differed with altitude. Protein content was highest at 2800 m (MX 94.28 g/100 g) and lowest at 1500 m (LX 91.28 g/100 g, *p* = 0.041), and fat content was highest at 1500 m (2.10 g/100 g) and lowest at 2800 m (1.73 g/100 g), giving a “high-protein, low-fat” profile at middle altitude. IMP was highest at 3500 m (HX 828.33 mg/100 g, *p* = 0.025) and UMP was lowest at middle altitude (MX 8.71 mg/100 g, *p* = 0.011). Total free amino acids, proline, lysine and arginine were higher at middle and high altitude (*p* < 0.05), whereas phenylalanine was lower at 2800 m (3.53 g/100 g, *p* = 0.008). For fatty acids, oleic acid was highest at low altitude (LX 0.39 g/100 g; *p* = 0.020) and DHA was higher at middle and high altitude (*p* = 0.024); among volatile flavour compounds, the relative content of hexane was markedly higher at middle and high altitude than at low altitude (*p* = 0.021) (Table 8). The direction of the altitudinal effects was not uniform across traits: protein and total free amino acids were highest at middle altitude, whereas IMP and hexane were highest at high altitude, and oleic acid was highest at low altitude. Grouping the same birds by maternal lineage did not resolve any consistent pattern, indicating that the meat-quality differences tracked altitude rather than maternal ancestry. Except for moisture, all compositional traits in Table 8 are expressed on a dry-matter basis; on this basis the sum of protein, fat and the remaining dry-matter components is consistent with 100%. After Benjamini–Hochberg correction across the 13 traits in Table 8, the associations with *p* ≤ 0.041 remained significant (q ≈ 0.036), whereas fat (*p* = 0.093) and moisture (*p* = 0.899) did not. Overall, the phenotypic data indicate that the three altitudinal groups differ in a coordinated way in muscle composition, whereas the mitochondrial lineages within them do not map onto those differences.

## 4. Discussion

### 4.1. Mitochondrial Variant Spectrum and Its Functional Significance

Ninety MT variant sites were identified, the great majority of which were transitions (83 of 90), in keeping with the transitional bias commonly observed in chicken mitochondrial DNA [21]. Most of these sites (85 of 90) were polymorphic within the population, indicating that the Muli Tibetan fragrant chicken carries considerable mitochondrial diversity despite its geographic isolation. Synonymous changes predominated among the coding variants (42 of 58), and the ratio of non-synonymous to synonymous coding variants (~0.38) was well below 1, consistent with the generally low coding diversity reported for chicken mitochondrial DNA [21]. Nonetheless, 16 non-synonymous substitutions were present, and their distribution was not random: they were concentrated in complex I (ND2, ND4L and ND5), complex IV (CO1 and CO2) and complex III (CYTB), the same complexes that have been implicated in high-altitude and high-energy-demand adaptation in other taxa [22,23]. In addition to the coding variants, the non-coding D-loop and rRNA regions harboured numerous polymorphic sites, which is expected because the control region is the most variable part of the animal mitochondrial genome and is the primary target used to define chicken haplogroups [21,24]. Similar non-synonymous mitochondrial variants have been associated with high-altitude adaptation in Tibetan populations [24], which supports the idea that variation in these complexes may be functionally relevant, although the specific amino-acid changes reported here differ from previously reported ones. Haplotype-based studies of high-altitude human and animal populations have likewise highlighted particular control-region and coding haplotypes as signatures of adaptation [24], and the finding of abundant variation in both the control region and the coding genes is consistent with this general pattern. At the population level, the mitochondrial genome of the Muli birds is therefore best described as variable but not exceptional: the level of diversity observed here is comparable to that reported for other Chinese indigenous chickens, in which numerous polymorphic sites are typically found in both the control region and the coding genes. The excess of synonymous over non-synonymous changes, together with the low overall number of amino-acid substitutions, indicates that most of the observed variation is neutral or nearly neutral with respect to protein function. Nevertheless, even a small number of non-synonymous changes can be functionally consequential if they occur at positions that influence subunit folding, proton translocation or the interaction between nuclear- and mitochondrial-encoded subunits, and the substitutions reported here therefore warrant follow-up rather than being dismissed as neutral.

Complex IV (cytochrome c oxidase) is the terminal oxidase of the respiratory chain and a key node in the regulation of oxygen utilization; variation in its subunits may alter the efficiency with which oxygen is reduced and thus affect performance under hypoxia [22]. Complex I (NADH:ubiquinone oxidoreductase) is the largest respiratory-chain complex and a major site of reactive oxygen species (ROS) production; substitutions in ND2, ND4L and ND5 could therefore influence both proton pumping and oxidative stress under low oxygen [22,23]. The substitutions detected—for example, ND5 nt14697 p.His543Asn and CYTB nt15535 p.Asp215Asn—are plausible candidate variants for hypoxia adaptation, but their functional consequences cannot be inferred from sequence data alone and will require structural modelling and functional validation. In addition, variation was also frequent in the D-loop and rRNA genes, consistent with the view that chicken mtDNA haplogroups are defined by both control-region and coding variation [21,24]. From a physiological standpoint, the demand for efficient oxygen use is highest in tissues with a large and fluctuating energy requirement, and skeletal muscle is one such tissue. If a coding variant slightly alters the catalytic rate or the proton-pumping stoichiometry of a respiratory-chain complex, the effect on the whole organism may be small under normoxia but larger under the sustained hypoxia of high altitude, where the margin between energy supply and demand is narrow. This reasoning underlies the interest in the non-synonymous sites reported here, although the present dataset cannot by itself establish such effects.

### 4.2. Maternal Lineages and Phylogenetic Placement

The 18 birds fell into three maternal lineages, with lineage A more frequent at low altitude and lineage B predominating at middle and high altitudes. This mild structure suggests that maternal lineages in the Muli population are partly, but not exclusively, associated with altitude, which is consistent with the broader finding that chicken mtDNA haplogroup distribution is shaped jointly by maternal origin, geography and demographic history rather than by a single environmental factor [21,25]. In the NJ tree, the population clustered close to *Gallus gallus gallus* and to Chinese local breeds, indicating that the maternal origin of Muli Tibetan fragrant chickens is dominated by haplogroups that are common in Chinese indigenous chickens and that may incorporate red-junglefowl ancestry [21,25]. Comparative mitogenomic studies of other Chinese local breeds—including the Zhuanghe Big-boned, Luhua, Longsheng Feng and Hetian chickens—have shown that each breed retains a characteristic but closely related maternal profile within the same broad haplogroup framework [26,27,28,29], and the placement of the Muli population is consistent with this pattern. The coexistence of different lineages within a single altitudinal group, and the sharing of lineages across altitudes, argues against strong altitude-specific selection on the maternal lineage and instead points to the movement of birds and to historical maternal diversity as the main determinants of lineage distribution. The coexistence of multiple lineages within groups, and their sharing across altitudes, suggests that the maternal mitochondrial genome of this population largely retains standing diversity rather than tracking altitude [21,25]. Similar demography-driven differentiation has been reported in local chicken populations of other regions, such as the Japanese long-tailed chicken [30] and Swedish local breeds [31], further supporting the view that breed structure in the mitochondrial genome reflects history rather than environmental selection. A further consideration is that chicken mitochondrial haplogroups are not evenly distributed across breeds and regions, and local breeds often retain mixtures of lineages as a result of historical gene flow, so the partial altitudinal structure observed here is best interpreted as a signature of maternal demography rather than of local selection [21,25]. Chicken mitochondrial lineages are classified into a small number of haplogroups (A–E and related lineages) whose frequencies differ among breeds and regions, and the placement of the Muli population close to *G. gallus gallus* and to Chinese local breeds is consistent with earlier reports on the maternal origin of Chinese indigenous chickens, which frequently belong to haplogroups that predominate in East Asia [21,25]. At a deeper level, the diversification of galliform birds is itself recent and rapid, and the phylogenetic relationships among closely related taxa can be difficult to resolve with short mitochondrial segments, so complete mitogenomes such as those used here provide more robust resolution [32]. It is also worth noting that samples from the same altitude group did not always share a lineage, and samples from different altitudes sometimes did, so altitude alone explains only part of the maternal structure. Such a pattern is typical of local breeds that are managed as village flocks, in which exchange of breeding birds, overlapping generations and small effective population sizes can produce a mosaic of maternal lineages that is not aligned with any single environmental variable. This mosaic structure also complicates the use of mitochondrial lineages as a stand-alone proxy for origin or adaptation, and it reinforces the need to combine mitochondrial markers with nuclear genomic data for population assignment.

### 4.3. Heteroplasmy and Its Potential Significance

Fifteen heteroplasmic sites were detected, and 16S rRNA nt3946 (G/C/A) was heteroplasmic in all 18 birds with strong read-level support. Because 16S rRNA is a structural component of the mitochondrial ribosome, variation at this site could in principle affect mitochondrial function [33,34]. A site that is heteroplasmic in every individual of a population is unusual and may reflect specific replication–segregation dynamics, relaxed purifying selection, or a balance between mutation and selection; alternatively, it may partly reflect sequencing depth and alignment characteristics at this position, so it should be confirmed by an orthogonal method [33,34]. By contrast, nt13450 in ND5 was heteroplasmic only in HX2 and HX5, indicating that heteroplasmy also varies among individuals. Heteroplasmy arises and is transmitted under the joint influence of the genetic bottleneck, the Balbiani body and mitophagy, and the tissue distribution of heteroplasmic variants can change with age and with metabolic state [33,35]. In the present study, the heteroplasmic sites were dominated by low-frequency alleles and were concentrated in the 16S rRNA and D-loop regions, which suggests that heteroplasmy in this population is largely confined to non-coding and translation-related regions rather than to protein-coding genes. Confirmation by Sanger sequencing, deeper targeted sequencing and family- or tissue-level transmission analysis will be necessary to establish the biological significance of these sites. From a mechanistic perspective, heteroplasmy at a functionally important site can shift the balance between normally folded and impaired respiratory complexes, and the outcome depends on the local allele load and on tissue-specific selection; in muscle, the large number of mitochondrial genomes per cell buffers modest shifts in allele frequency but can also propagate them clonally as fibres age and remodel [33,34]. The transmission of functional, wild-type mitochondria and of the fittest mtDNA to the next generation involves selective mechanisms that act during oogenesis [35], and the persistence of low-frequency variants in the adult birds suggests that such filtering is incomplete. Two further observations deserve comment. First, the fact that the same heteroplasmic site appeared in all 18 birds suggests that it is either ancient and stably maintained or subject to recurrent mutation, and distinguishing between these possibilities would require sequencing of related breeds and of maternal pedigrees. Second, the coding heteroplasmies detected, such as nt13450 in ND5, were present in only a subset of individuals and at moderate allele frequencies, which is more consistent with recent mutation or with incomplete segregation than with long-term balancing selection. Both patterns underline that heteroplasmy in this population is dynamic and heterogeneous among individuals. It should also be noted that the detection of heteroplasmy is strongly depth-dependent, and sites that are heteroplasmic at low frequency in high-depth samples may be scored as homoplasmic, or missed entirely, in shallower ones. This means that the absolute number of heteroplasmic sites reported here (15) is best regarded as a lower bound, and that comparisons of heteroplasmy burden among studies must account for differences in sequencing depth.

### 4.4. mtDNA Copy Number

Relative mtDNA copy number varied more than fivefold among individuals but did not differ significantly among altitude groups and showed no consistent altitudinal trend. mtDNA copy number is regulated by tissue energy demand and by oxidative stress, and skeletal muscle is a high-copy-number tissue with substantial individual variation, which is consistent with the present observations [20]. The results suggest that high-altitude adaptation in this breed is not achieved by simply increasing the amount of mtDNA; instead, adaptation may operate through the regulation of mitochondrial function, such as the efficiency of OXPHOS and the control of ROS [24,36]. Because the copy-number estimates were derived from whole-genome sequencing depth, they are relative rather than absolute, and absolute quantification would require quantitative PCR or digital PCR [20]. Because copy number can vary with muscle fibre type, age and metabolic state, part of the inter-individual variation observed here may also reflect physiological differences, and future work should combine copy-number measurement with fibre-type and oxidative-stress indicators [20]. It is important to note that the copy-number values reported here should not be over-interpreted. Whole-genome sequencing depth is a convenient but indirect measure of mitochondrial content, and it is influenced by factors such as the proportion of repeat and organelle-derived reads, library composition and alignment efficiency. Even so, the absence of a consistent altitudinal trend is notable, because it argues against a simple model in which adaptation to hypoxia is achieved by uniformly increasing mitochondrial biomass in muscle. Instead, adaptation may be achieved by tuning the quality rather than the quantity of the mitochondrial population—for example, by adjusting respiratory-chain stoichiometry, by remodelling the balance between complexes, or by altering the efficiency of substrate oxidation—and such adjustments would not necessarily be visible in a copy-number measurement.

### 4.5. Phenotypes, Meat Quality and the Mitochondrial Connection

Heart and lung weights increased with altitude, consistent with the adaptive response of Tibetan chickens to the high-altitude environment [37]. Meat quality, in contrast, changed mainly with altitude rather than with maternal lineage: the middle-altitude group combined the highest protein and the lowest fat content, giving a favourable “high-protein, low-fat” profile; the high-altitude group had the highest IMP content, which is the principal umami compound of chicken meat and an important determinant of flavour [38]; and the low-altitude group had the highest oleic acid but lower DHA, whereas DHA was higher at middle and high altitude. These differences are consistent with metabolic adjustments reported in Tibetan chickens and with the slow-growing character of the breed [37,39]. Studies of Tibetan chicken transcriptomes have reported widespread changes in genes related to energy metabolism and hypoxia signalling between high- and low-altitude birds [40], and a liver transcriptome study of hypoxia response similarly identified coordinated shifts in mitochondrial and metabolic pathways [39], which together suggest that the altitudinal differences in muscle composition observed here are underlain by broad transcriptional remodelling rather than by mitochondrial sequence variation alone. Notably, none of these traits showed a consistent relationship with maternal lineage, which supports the interpretation that meat quality and other high-altitude phenotypes are driven principally by nuclear genes and the environment rather than by mitochondrial sequence variation. In addition, amino-acid composition was generally stable across groups, suggesting that the amino-acid profile of breast muscle was only mildly affected by altitude [37]. From the perspective of product quality, the combination of high protein, low fat and high IMP at middle altitude is consistent with the slow-growing, energy-metabolism-active character of Tibetan chickens and with earlier observations that IMP accumulates to high levels in this breed [38], while the altitudinal shifts in oleic acid and DHA indicate that environmental factors influence the fatty-acid profile of breast muscle; the absence of a consistent association with maternal lineage suggests that these quality traits are controlled mainly by nuclear genes and by rearing environment rather than by mitochondrial sequence variation [37,38]. The absence of a relationship between meat quality and maternal lineage is one of the more informative negative results of this study. It indicates that, within the range of mitochondrial variation present in the Muli population, the maternal genome does not by itself determine the muscle phenotypes measured here, and that any mitochondrial contribution to meat quality is likely to be small relative to the effects of nuclear genotype, age, diet and rearing environment. This conclusion is consistent with the broader observation that economically important traits in poultry are usually polygenic and are only weakly influenced by individual mitochondrial variants. Practically, this means that selection for meat quality should rely on phenotypic and nuclear-genomic information, while mitochondrial data are most useful for traceability and for monitoring maternal diversity.

### 4.6. Limitations

The present study has several limitations. First, the sample size (n = 18) and the sequencing depth (approximately 4x) were limited, so the estimates of heteroplasmy and copy number are preliminary and require validation by targeted deep sequencing. Second, structural- and copy-number-variant detection is insensitive for the mitochondrial genome, so the apparent absence of MT SVs should be confirmed by higher-depth or long-read sequencing. Third, the association between mitochondrial variation and phenotype was exploratory, and the functional effects of the candidate non-synonymous variants remain to be tested experimentally. Because each altitude was represented by a single rearing site, altitudinal effects cannot be fully separated from site-specific environmental effects, and the observed differences should therefore be interpreted as altitudinal associations rather than as direct evidence of altitude adaptation. Absolute quantification of mtDNA copy number by qPCR or digital PCR, and orthogonal validation of heteroplasmy by Sanger sequencing or targeted deep sequencing, were not performed; the copy-number and heteroplasmy results should therefore be regarded as relative and preliminary. Finally, expanding the sample size, the altitudinal range and the number of rearing environments, and integrating nuclear genomic and functional evidence, will be needed to confirm these findings.

### 4.7. Synthesis and Implications

Overall, the mitochondrial genome of Muli Tibetan fragrant chickens carries substantial standing variation, most of it synonymous or non-coding, with maternal lineages only partly structured by altitude, heteroplasmy common and copy number stable across altitudes. Meat quality, in contrast, changed markedly with altitude but was unrelated to maternal lineage. This combination suggests that high-altitude adaptation in this breed is driven mainly by nuclear genes and the environment, whereas the mitochondrial genome contributes standing diversity and provides useful maternal markers. These markers, together with the candidate variants identified here, can support breed authentication, traceability and conservation planning, and the altitude of rearing should be considered when producing premium-quality Tibetan fragrant chicken products.

## 5. Conclusions

The breast-muscle mitochondrial genome of Tibetan fragrant chickens from Muli County harbours abundant sequence variation (90 sites, of which 16 are non-synonymous) and heteroplasmy (15 sites, with 16S rRNA nt3946 heteroplasmic in every bird). The birds formed three maternal lineages (A, B and C) with only mild altitudinal differentiation, and their maternal origin closely resembles that of Chinese local chickens. Relative mtDNA copy number did not differ among altitude groups. Meat quality, including high protein and low fat at 2800 m and the highest IMP at 3500 m, was associated mainly with altitude rather than with maternal lineage. Taken together, these findings provide a preliminary characterization of mitochondrial variation in this breed: the observed phenotypic differences were associated mainly with altitude of rearing rather than with maternal mitochondrial lineage, and the mitochondrial genome appears to contribute standing genetic diversity together with maternal markers that may be useful for breed authentication and conservation. These interpretations are exploratory and require confirmation in larger studies with independent sites.

## Figures and Tables

**Table 1:** Sequencing and mapping quality of the 18 samples (n = 18).

Index	Range	Mean
Q30/%	95.96–96.75	96.30
GC content/%	43.34–45.09	44.03
Mapping rate/%	62.94–98.94	95.03
Mean depth/×	2.99–4.69	3.97
Coverage ≥ 1×/%	71.95–94.36	88.51
Coverage ≥ 4×/%	9.33–49.22	32.23

**Table 2:** Regional distribution and effect of the 90 MT variant sites.

Category	Count
Coding (CDS)	58
synonymous	42
non-synonymous	16
D-loop/non-coding	20
rRNA	10
tRNA	2
Total	90

**Table 3:** Non-synonymous MT variants and their amino-acid substitutions (n = 16).

Gene	Site	Change	Substitution	No. of Birds
ND2	nt5274	A > G	p.Ser12Gly	1
ND2	nt5718	T > G	p.Ser160Ala	10
ND2	nt5743	T > C	p.Met168Thr	1
ND2	nt5928	C > A	p.Leu230Met	18
CO1	nt7030	T > C	p.Val129Ala	1
CO1	nt7530	C > G	p.Arg296Gly	18
CO1	nt8070	T > C	p.Phe476Leu	6
CO2	nt8464	T > C	p.Met45Thr	3
CO2	nt8787	A > G	p.Ile153Val	3
ND4L	nt11364	G > A	p.Ala57Thr	1
ND5	nt13219	T > C	p.Ile50Thr	1
ND5	nt13450	T > C	p.Ile127Thr	2 (hetero)
ND5	nt14437	G > A	p.Arg456Gln	1
ND5	nt14692	T > C	p.Leu541Pro	1
ND5	nt14697	C > A	p.His543Asn	10
CYTB	nt15535	G > A	p.Asp215Asn	4

**Table 4:** Distribution of maternal lineages across altitudes.

Lineage	LX (1500 m)	MX (2800 m)	HX (3500 m)	Total
A	4	1	1	6
B	1	5	4	10
C	1	0	1	2
Total	6	6	6	18

**Table 5:** Read-level evidence for the MT nt3946 site (AD = ref, allele 1, allele 2).

Sample	GT	DP	AD (G,C,A)	Sample	GT	DP	AD (G,C,A)
HX1	1/2	45	6,23,14	LX1	1/2	810	57,391,352
HX2	0/1	41	8,16,12	LX2	0/1	33	10,9,10
HX3	1/2	69	14,23,25	LX3	1/2	217	20,110,83
HX4	0/1	64	16,20,20	LX4	1/2	165	19,82,53
HX5	1/2	79	13,27,31	LX5	0/1	63	10,18,24
HX6	1/2	1035	95,536,368	LX6	1/1	732	69,358,299
MX1	0/1	36	13,9,9	MX4	1/1	46	7,22,13
MX2	0/2	15	3,2,6	MX5	0/1	62	12,23,23
MX3	0/1	55	17,11,16	MX6	0/1	51	6,14,18

**Table 6:** Relative mtDNA copy number of each sample (MT depth/nuclear depth).

Sample	MT Depth	Nuclear Depth	CN	Sample	MT Depth	Nuclear Depth	CN
LX1	34,258	4.69	7304	MX1	17,943	4.32	4153
LX2	29,613	4.41	6715	MX2	8512	4.28	1989
LX3	23,114	4.20	5503	MX3	30,595	4.28	7148
LX4	25,997	3.51	7407	MX4	13,250	3.44	3852
LX5	36,398	3.77	9655	MX5	44,100	3.84	11,484
LX6	38,253	4.09	9353	MX6	33,731	3.44	9806
HX1	27,187	2.99	9093	HX4	30,787	4.12	7473
HX2	26,585	3.73	7127	HX5	38,559	3.91	9862
HX3	40,931	3.82	10,715	HX6	30,771	4.62	6660

**Table 7:** Key phenotypes by altitude and by maternal lineage (means).

Trait	By Altitude L/M/H	By Lineage A/B/C
Heart/g	8.54/8.23/11.02	8.94/9.41/9.48
Lung/g	10.01/7.91/11.47	10.66/9.49/8.74
Body weight/kg	1.28/1.31/1.39	1.26/1.34/1.43
IMP/(mg·100 g^−1^)	775.2/778.5/828.3	776.2/804.5/795.0

**Table 8:** Main meat quality traits of breast muscle at three altitudes (mean ± SD, n = 6). Moisture is expressed on a fresh-weight basis; all other traits are expressed on a dry-matter (DM) basis.

Trait	LX (1500 m)	MX (2800 m)	HX (3500 m)	*p*
Moisture/(g·100 g^−1^ fresh weight)	73.12 ± 0.88	72.93 ± 1.23	73.37 ± 1.19	0.899
Protein/(g·100 g^−1^ DM)	91.28 ± 1.43	94.28 ± 2.62	92.93 ± 1.15	0.041
Fat/(g·100 g^−1^ DM)	2.10 ± 0.24	1.73 ± 0.21	2.00 ± 0.37	0.093
Total free amino acids/(% DM)	3.17 ± 0.10	3.42 ± 0.11	3.31 ± 0.12	0.009
Phenylalanine/(g·100 g^−1^ DM)	4.96 ± 0.10	3.53 ± 0.10	4.78 ± 0.60	0.008
Proline/(g·100 g^−1^ DM)	2.35 ± 0.08	2.79 ± 0.08	2.42 ± 0.18	0.008
Arginine/(% DM)	0.07 ± 0.01	0.09 ± 0.01	0.09 ± 0.01	0.014
Lysine/(% DM)	0.06 ± 0.01	0.08 ± 0.01	0.08 ± 0.01	0.032
IMP/(mg·100 g^−1^ DM)	775.17 ± 33.61	778.50 ± 44.97	828.33 ± 14.94	0.025
UMP/(mg·100 g^−1^ DM)	11.72 ± 1.32	8.71 ± 1.99	11.47 ± 0.97	0.011
Oleic acid/(g·100 g^−1^ DM)	0.39 ± 0.06	0.27 ± 0.06	0.31 ± 0.05	0.020
DHA/(g·100 g^−1^ DM)	0.04 ± 0.01	0.06 ± 0.02	0.06 ± 0.01	0.024
Hexane/(% DM)	5.76 ± 4.31	17.78 ± 6.61	15.52 ± 12.62	0.021

## Data Availability

The raw sequencing data generated in this study are being deposited in a public sequence repository, and the accession numbers will be provided at the revision stage. Other data are available from the corresponding authors on reasonable request.

## Abbreviations

MT, mitochondrial; mtDNA, mitochondrial DNA; CDS, coding sequence; VAF, variant allele frequency; CN, copy number; IMP, inosine monophosphate; DHA, docosahexaenoic acid; LX/MX/HX, low/middle/high altitude; OXPHOS, oxidative phosphorylation; SV, structural variant; CNV, copy-number variant.
